# Antioxidant effects of Emblica officinalis and Zingiber officinalis on arsenic and lead induced toxicity on Albino rats

**DOI:** 10.6026/97320630017295

**Published:** 2021-02-28

**Authors:** Mohd Fazal, Vishnu Priya Veeraraghavan, Bushra Tahreen, Selvaraj Jayaraman, R Gayathri

**Affiliations:** 1 Department of Biochemistry, Saveetha Dental College, Saveetha Institute of Technical and Medical Sciences, Saveetha University, Chennai, India; 2 Department of Anatomy Government Medical College Siddipet, Telangana, India; 3 Department of Dentistry, International Dental Care, Banjara Hills, Hyderabad, Telangana, India

**Keywords:** Antioxidant enzymes, nephrotoxicity, hepatotoxicity, oxidative stress

## Abstract

It is of interest to document the effect of Emblica officinalis (E. officinalis) and Zingiber officinalae (Z. officinalae) leaf extract on reactive oxygen species, antioxidant potential changes in arsenic and lead-induced toxicity in male rats. We used 8 groups of adult male Wistar rats with 1 control group for this study. The animals were divided into Group I: Control and Group II: Lead and sodium arsenite induced rats (animals were induced for metal toxicity by the combined administration of arsenic (13.8 mg/ kg body weight) and lead (116.4 mg/kg body weight). These doses were administered by gastric intubation during 14 consecutive days using known standard procedures. Arsenic and lead induced rats treated with ethanolic extract of Emblica officinalis (60 mg/kg body weight/day, orally for 45 days) are group III rats. Group IV animals are arsenic and lead induced rats treated orally with ethanolic extracts of E. officinalis (120 mg/kg body weight/day for 45 days). Group V animals are arsenic and lead induced rats treated orally with ethanolic extracts of Z. officinalae (60 mg/kg body weight/day for 45 days). Group VI animals are arsenic and lead induced rats orally treated with ethanolic extracts of Zingiber officinalis (120 mg/kg body weight/day for 45 days). Group VII animals are arsenic and lead induced rats treated orally with ethanolic extracts of E. officinalis and Z. officinalae (60 + 60 mg/kg body weight/day for 45 days). Group VIII animals are arsenic and lead induced rats treated orally with ethanolic extracts of E. officinalis and Z. officinalae (120 + 120 mg/kg body weight/day, orally for 45 days). Normal Control animals were treated orally with ethanolic extracts of E. officinalis (120mg/kg body weight) + Z. officinalae (120mg/kg body weight) for 45 days. The control and experimental animals were then subjected to analysis for oxidative stress markers such as H2O2, *OH, and lipid peroxidation (LPO), antioxidant enzymes in addition to liver and kidney function markers. Results: Arsenic and lead induced rats showed a significant increase in the levels of reactive oxygen species (H2O2, OH* and LPO) with concomitant alterations in the renal and liver tissues. However, enzymic and non-enzymic antioxidant levels were decreased. Nevertheless, an oral effective dose of E. officinalis and Z. officinalae (120 + 120 mg/kg body weight/day increased the antioxidant enzymes and retrieved the altered levels of ROS and LPO that were induced by arsenic and lead. Thus, we show that E. officinalis and Z. officinalae leaf extract exhibits nephroprotective and hepatoprotective role through the restoration of reactive oxygen species and antioxidant enzymes in the kidney and liver tissue of Arsenic and Lead-induced nephrotoxicity and hepatotoxicity in rats. Hence, E. officinalis and Z. officinalae leaf extract are potential therapeutic options for the treatment of metal toxicity-induced kidney and liver diseases.

## Background

Metals are found naturally in the environment and their compositions vary among different localities, resulting in spatial variations of surrounding concentrations. Distribution of these heavy metals in the atmosphere is affected by various environmental factors [1]. Heavy metals are usually referred to as those metals, which possess a specific density of more than 5 g/cm3 and adversely affect the habitat and living organisms [2]. Arsenic and lead enter the surroundings by natural means and through human activities. Various routes of arsenic and lead exposure include soil erosion, natural weathering of the earth's crust, mining, industrial effluents, urban runoff, sewage discharge, insect or disease control agents applied to crops, and many others [3]. Earlier study states that oxidative deterioration of biological macromolecules is primarily due to binding of heavy metals to the DNA and nuclear proteins [4]. Indian gooseberry (Phyllanthus emblica officinalis) extracts, has been used in traditional medicine to treat symptoms ranging from constipation to cancer treatment for centuries in the Indian system of medicine [5]. E. officinalis has been shown to be a potent free radical scavenging agent, thereby preventing carcinogenesis and mutagenesis. A dose of 100 mg/Kg body weight has shown to reduce the incidence of tumour by approximately 60% [6,7]. Ginger (Zingiber officinale Roscoe) is also another natural dietary component generally used in complementary and alternative medicine (CAM). Various ginger and ginger leaf extracts have been reported positive response in controlling cancer proliferation [8]. However, therapeutic effects of E.officinalis and Z. officinalae on metal-toxicity on multiple organ damage have not been report. Therefore, it is of interest to document the effect of E. officinalis and Z. officinalae in arsenite and lead-induced toxicity in male albino rats.

## Methodology

All chemicals and reagents used in the present study were molecular and analytical grade; and they were purchased from Sigma Chemical Company, St. Louis, MO, USA; Amersham Biosciences, Little Chalfont, Buckinghamshire, United Kingdom; and Sisco Research Laboratories, Mumbai, India; Arsenic and Lead was purchased from Sigma Chemicals Company, USA. Biochemical assay kits used in the present study were purchased from Spinreact, Spain.

## Animals:

Animals were maintained as per the guidelines and protocols approved by the Institutional Animal's Ethics Committee and by the regulatory body of the government (IAEC No: BRULAC/SDCH/SIMATS/IAEC/09-2018/009). Healthy male albino rats of Wistar strain (Rattus norvegicus) weighing 180-210 g (150–180 days old) were used in this study. Animals were obtained and maintained in clean polypropylene cages under specific humidity (65 ± 5%) and temperature (27 ± 2 °C) with constant 12 h light and 12 h dark schedule at Biomedical Research Unit and Lab Animal Center (BRULAC), Saveetha Dental College and Hospitals, Saveetha Institute of Medical and Technical Sciences, Chennai - 600 077. They were fed with standard rat pelleted diet (Lipton India, Mumbai, India), and clean drinking water was made available ad libitum.

## Experimental design:

We used 8 groups of adult male Wistar rats with 1 control group for this study. The animals were divided into Group I: Control and Group II: Lead and sodium arsenite-induced rats (animals were induced for metal toxicity by the combined administration of arsenic (13.8 mg/kg body weight) and lead (116.4 mg/kg body weight). These doses were administered by gastric intubation during 14 consecutive days using known standard procedures. Arsenic and lead induced rats treated with ethanolic extract of Emblica officinalis (60 mg/kg body weight/day, orally for 45 days) are group III rats. Group IV animals are arsenic and lead induced rats treated orally with ethanolic extracts of E. officinalis (120 mg/kg body weight/day for 45 days). Group V animals are arsenic and lead induced rats treated orally with ethanolic extracts of Z. officinalae (60 mg/kg body weight/day for 45 days). Group VI animals are arsenic and lead induced rats orally treated withethanolic extracts of Zingiber officinalis (120 mg/kg body weight/day for 45 days). Group VII animals are arsenic and lead inducedrats treated orally with ethanolic extracts of E.officinalis and Z. officinalae (60 + 60 mg/kg body weight/day for 45 days). Group VIII animals are arsenic and lead induced rats treated orally with ethanolic extracts of E. officinalis and Z. officinalae (120 + 120 mg/kg body weight/day, orally for 45 days). Normal Control animals were treated orally with ethanolic extracts of E. officinalis (120mg/kg body weight)+ Z.officinalae (120mg/kg body weight) for 45 days.

## Biochemical Estimation:

Assessment of liver function markers

Liver function markers such as alanine aminotransferase (ALT), aspartate aminotransferase (AST), alkaline phosphatase (ALP) and bilirubin were measured by kit method as per the manufacturer instructions and results were expressed as IU/ml.

## Assessment of kidney function markers:

Kidney function markers (urea and creatinine) were measured using biochemical-assay kits procured from Spinreact, Spain using Semi-Automated Biochemistry Analyser System (Coralab 3000) and results for thesame were expressed as mg/dl.

## Assessment of serum lipid markers:

Serum cholesterol (CHO), triglyceride (TG), low-density lipoproteins (LDL), high-density lipoproteins (HDL) were assessed using assay kits purchased from spin react Spain. Results were expressed as mg/dl.

## Measurement of oxidative stress markers:

Lipid peroxidation (LPO) was measured by a previously published method of Devasagayam and Tarachand [9]. Hydrogen peroxide generation (H2O2) was assessed by the spectrophotometric method of Pick and Keisari [10]. Hydroxyl radical (OH*) production was quantified by the method of Puntarulo and Cederbaum [11].

## Antioxidant Enzymes:

Superoxide dismutase (SOD) activity was assessed by the method of Marklund and Marklund [12]. Catalase activity (CAT) was assessed as per the method of Sinha [13]. Glutathione (GSH) peroxidase (GPx) levels were assessed by the method of Rotruck et al. [14] GSH -S-transferase (GST) activity was assessed by the method of Habig et al. [15] GSH reductase (GR) was assessed as per the method of Staal et al. [16] and reduced GSH levels were measured by the method of Moron et al. [17].

## Statistical Analysis:

The data were subjected to statistical analysis using one-way analysis of variance and Duncan's multiple range test to assess the significance of individual variations between the control and treatment groups using a computer-based software (GraphPad Prism version 5). In Duncan's test, the significance was considered at the level of p< 0.05.

## Results:

**Figure 1:**
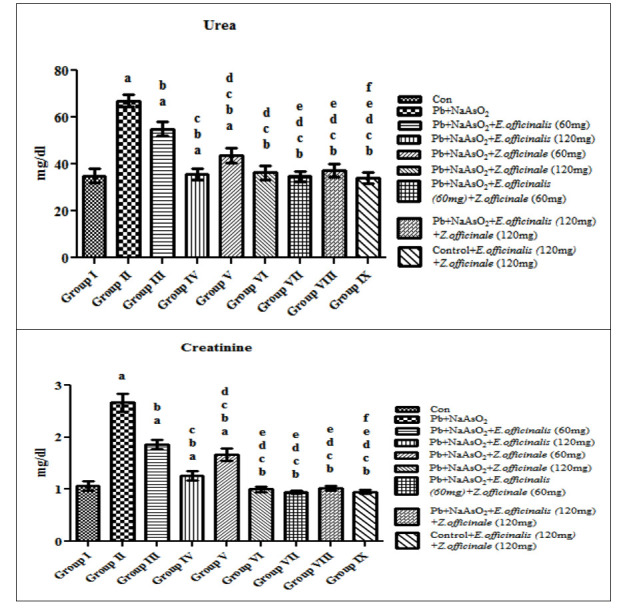
Effect of E. officinalis and Z. officinale on kidney function makers (Urea and Creatinine) in lead-arsenic-induced toxicity in adult male rat. Each bar represents mean ± SEM of 6animals. Significance at p< 0.05, a-compared with control, b-compared with Lead-Arsenic toxicity, c-compared with Lead-Arsenic toxicity rats treated with 60mg E.officinalis; d- compared with Lead-Arsenic toxicity rats treated with 120mg E. officinalis; e- compared with Lead-Arsenic toxicity rats treated with 60mg Z.officinale; f-compared with Lead-Arsenic toxicity rats treated with 60 E.officinalis+60mg Z. officinale.

Arsenic and Lead-administered rat showed elevated levels of Urea and Creatinine when compared with control. However, the body weight of the animals was significantly reduced due to Arsenic and Lead induction (Figure 1a-b). Treatment with extract of Emblica offcinalis and Zingiber officinalis at the doses of 60mg/kg body weight did not show any effective reduction on the elevated levels of kidney markers and could not increase the body weight of the animal to that of control groups whereas Combined treatment with extract of Emblica offcinalis and Zingiber officinalis120 mg/kg body weight dose significantly normalized the kidney function markers and improved the body weight which altered by Arsenic and Lead induction. Control rats treated with Emblica officinalis and Zingiber officinalis extract did not showed any significant change showing the effective dose of leaf extract does not have any toxicity.

**Figure 2:**
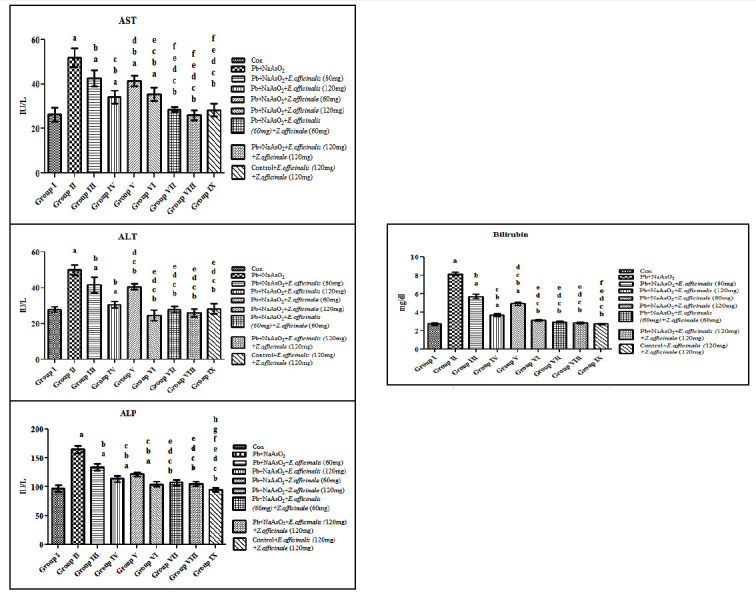
Effect of E.officinalis and Z.officinale on liver function markers (AST, ALT, ALP and Total Bilirubin) in lead-arsenic-induced toxicity in adult male rat. Each bar represents mean ± SEM of 6 animals. Significance at p< 0.05, a-compared with control, b-compared with Lead-Arsenic toxicity, c-compared with Lead-Arsenic toxicity rats treated with 60mg E.officinalis; d- compared with Lead-Arsenic toxicity rats treated with 120mg E.officinalis; e- compared with Lead-Arsenic toxicity rats treated with 60mg Z.officinale; f-compared with Lead-Arsenic toxicity rats treated with 60 E. officinalis+60mg Z.officinale. Total and direct bilirubin levels were significantly increased in Arsenic and Lead-induced rats whereas administration of Emblica officinalis and Zingiber officinalis extract normalized the bilirubin levels to that of the control. Control rats treated with Emblica officinalis and Zingiber officinalis extract did not showed any significant change in the bilirubin levels (Figure 2d). Increased levels of Total cholesterol, TG, and LDL cholesterol (LDLC) but low HDL cholesterol (HDLC) were observed in the Arsenic-Lead induced animals. Emblica officinalis and Zingiber officinalis leaf extract treated experimental rats significantly alleviated dyslipidaemia to that control (Figure a-c).

Liver function markers (ALT, AST, and ALP) were significantly elevated in Arsenic and Lead-induced Toxicity in rats when compared to control. Treatment with extract of Emblica offcinalis and Zingiber officinalis at the doses of 60mg/kg body weight could not reduce the enzyme levels to that of the control [Figure 2a-c]. However, Combined treatment with extract of Emblica offcinalis and Zingiber officinalis120 mg/kg body weight dose significantly restored the liver function markers to control level. Control rats treated with Emblica officinalis and Zingiber officinalis extract did not showed any significant change showing the effective dose of leaf extract does not have any toxicity.

Compared to control, the H2O2, OH*, and LPO in kidney were significantly raised in Arsenic-Lead induced rats. Treatment with Emblica officinalis and Zingiber officinalis leaf extract notably brought down the rise in hydrogen peroxide, OH*, and LPO (Table 1). Compared to control, the H2O2, OH*, and LPO in kidney were significantly raised in Arsenic-Lead induced rats. Treatment with Emblica officinalis and Zingiber officinalis leaf extract notably brought down the rise in hydrogen peroxide, OH*, and LPO (Table 2). There was a marked decrease observed in antioxidants enzymes (SOD, CAT, GPx, GST, GR, GSH) in the kidney of Arsenic-Lead induced Toxicity group compared to control. Emblica officinalis and Zingiber officinalis efficiently increased the level of antioxidant enzymes compared to the induced group due to the presence of antioxidants in the extract (Table 3). There was a marked decrease observed in antioxidants enzymes (SOD, CAT, GPx, GST,) in the kidney of Arsenic-Lead induced Toxicity group compared to control. Emblica officinalis and Zingiber officinalis efficiently increased the level of antioxidant enzymes compared to the induced group due to the presence of antioxidants in the extract (Table 4).

## Discussion:

It is of interest to determine the effects of Lead-Arsenic induced oxidative damage and role of Emblica officinalis and Zingiber officinalis in Lead-Arsenic methylation metabolism. Arsenic is one of the most important naturally occurring toxic metalloid causing concern from both ecological and individual health viewpoints [18]. Arsenic ranks twentieth among the elements in abundance in earth's crust and its inorganic forms such as arsenite and arsenate compounds are lethal to the environment and living creatures. Exposure of arsenic to humans may occur by drinking contaminated water, cigarettes, industrial source, food or from unintended sources. Among the various routes of exposure drinking water is one of the largest sources of arcenicosis. Contamination of drinking water occurs by the use of arsenical pesticides, natural mineral deposits or inappropriate disposal of arsenical chemicals. Intentional consumption of arsenic in case of suicidal attempts or accidental consumption by children may also result in cases of acute poisoning [18,19]. Arsenic primarily affects the sulfhydryl group of cells causing malfunctioning of cell respiration, cell enzymes and mitosis [20]. Arsenic (usually as arsenic trioxide, As2O3) is well known as a poison and has been discovered to be a carcinogen in humans. Extreme levels of arsenic exposure occur mainly in localities where arsenic is found naturally i.e. soils, rocks, and water, and in workplaces, near or in risky waste sites. Chronic exposure to inorganic arsenic can lead to cancer of the skin, lungs, bladder, and liver if the exposure is through ingestion. Lung cancer can occur if exposure is by inhalation [21,22]. Low levels of arsenic exposure for prolonged periods of time can give rise to discoloration of the skin and the appearance of small corns or warts [23]. High levels of arsenic exposure can cause death. Lead (Pb) is ubiquitous and naturally occurring bluish grey metal found in small amount in earth crust and has been recognized as a major health hazard for living organisms on the earth. High malleability, low melting point, ductility softness and resistance to corrosion, are some of the unique properties of lead. Lead has been used in different industries like automobiles, paint, ceramics, plastics, etc. The sources of lead exposure include mainly industrial processes, food and smoking, drinking water and domestic sources. Lead remains present in the atmosphere (water, soil or industrial products) because of its persistent nature. Worldwide reports suggest that children are more susceptible to lead as gastrointestinal absorption is higher and blood lead levels lower than 10 µg/dl may also cause serious manifestations like slowed cognitive development and neuropsychological disorders [24,25,26]. The liver function marker enzymes (ALT, AST, and ALP) and the bilirubin levels in serum are long-standing indicator of hepatotoxicity [27]. We observed a significant increase in the levels of ALT, AST, and ALP and total bilirubin in Arsenic-Lead administered experimental rats which suggesting their cellular leakage and loss of functional integrity of cell membrane in liver. E.officinalis and Z.officinal ethanolic leaf extract treatment significantly decreased the liver function marker enzymes and bilirubin toward the respective normal level that is an indication of stabilization of plasma membrane as well as repair of hepatic tissue damage caused by the Arsenic-Lead administration. Karadka Ramdas Thilakchand et al. [28] demonstrated that hepatoprotective actions of amla is mediated by its free radical scavenging, antioxidant, anti-inflammatory, modulation of the xenobiotic detoxification process and lipid metabolism. Urea and creatinine levels in serum are crucial biomarkers of renal function in human and animal studies [29]. Data from the present study revealed that Arsenic-Lead administration caused a significant increase in urea and creatinine and levels may be due to decreased glomerular filtration rate, which is an indication of loss of renal function. This may attribute to oxidative stress-induced cellular damage induced by Arsenic-Lead administration. Considering, treatment with E. officinalis and Z. officinale ethanolic leaf extract significantly restored the altered levels of urea and creatinine, which may be due to the repair of renal tissue damage mediated by the antioxidants potential of extracts. We also observed that Arsenic-Lead administration significantly increased the level of total cholesterol (TC) and that may be due to increased activity of lipogenic enzymes mediated by Arsenic-Lead [30]. An elevated levels of FFA, LDL, and VLDL also observed in Arsenic-Lead induced toxicity in rats that may be due to the stimulation of sympathetic adrenal system by Arsenic-Lead leading to increased secretion of catecholamines resulting in increased lipolysis and synthesis of TG-rich lipoproteins [32,33]. A concomitant decrease in the level of HDL was also observed in Arsenic-Lead administered rats as a result of decreased activity of lipoprotein lipase and CHO acyltransferase due to Arsenic-Lead administration. E.officinalis and Z. officinale ethanolic leaf extract treatment significantly decreased the elevated levels of TC, FFA, LDL, and VLDL and concomitantly increased the level of HDL in experimental rats which may due to the presence of a biologically active compound that modulates the lipid metabolism by decreasing the oxidative stress. Moreover, the finding of the current study indicates that the exposure of Arsenic-Lead results in elevated levels of LPO, H2O2, and *OH that may be due to enhanced activity of Either redox-active or redox-inactive metals may cause an increase in production of reactive oxygen species (ROS) such as hydroxyl radical (HO.), superoxide radical (O2.-) or hydrogen peroxide (H2O2). Enhanced generation of ROS can overwhelm cells' intrinsic antioxidant defences, and result in a condition known as "oxidative stress". Cells under oxidative stress display various dysfunctions due to lesions caused by ROS to lipids, proteins and DNA [34]. An elevated levels of LPO is associated with a wide variety of toxic effects, including decreased membrane fluidity and function, impaired functions of the mitochondria and Golgi apparatus and inhibition of enzymes which results in tissue damage and organ dysfunction [35]. We also found a significant decline in antioxidant enzymes includes SOD, CAT, GPx, GST, GR, and GSH was also observed in Arsenic-Lead administered rats. This may be due to increased utilization of antioxidants to scavenge excess ROS induced by Arsenic-Lead. The treatment with E. officinalis and Z. officinale ethanolic leaf extract significantly decreased the level of ROS with elevating the antioxidant enzymes in Arsenic-Lead induced nephrotoxic rats that may be due to the presence of antioxidants in the extract.

## Conclusion

The kidney (urea and creatinine) and liver function markers (ALP, AST, ALT, and BR), and lipid markers (LDL, HDL, VLDL, TG, and FFA) were found to be altered in Arsenic-Lead induced rats. Antioxidants (SOD, CAT, GPx, GST, and GR,) are altered due to Arsenic-Leadinduced toxicity. Treatment with the effective dose (120mg/kg b.wt) of E.officinalis and Z.officinaleleaf extract significantly improved the altered levels of the biochemical profiles. Thus, E.officinalis and Z.officinale exhibit protective role through the restoration of serum biochemical profiles and antioxidant enzymes in the kidney and liver tissue of arsenic-lead induced toxicity in male rats. Further studies are needed to elucidate the exact mechanisms of protective role of E. officinalis and Z. officinale to ascertain its potential.

## Figures and Tables

**Table 1:** Effect of E.officinalis and Z. officinale on oxidative stress (H2O2&OH*) and lipid peroxidation (LPO) in the kidney tissue of lead-arsenic-induced toxicity in adult male rat. Each bar represents mean ± SEM of 6 animals. Significance at p< 0.05, a-compared with control, b-compared with Lead-Arsenic toxicity, c-compared with Lead-Arsenic toxicity rats treated with 60mg E.officinalis; d- compared with Lead-Arsenic toxicity rats treated with 120mg E.officinalis; e-compared with Lead-Arsenic toxicity rats treated with 60mg Z.officinale; f-compared with Lead-Arsenic toxicity rats treated with 60 E.officinalis+60mg Z.officinale.

Parameters	Group I	Group II	Group III	Group IV	Group V	Group VI	Group VII	Group VIII	Group IX
H2O2	1±0.111	2.9±0.27a	2.0±0.10ab	1.8±0.17abc	1.2±0.57bcd	1±0.05bcd	1.2±0.05bcd	1.13±0.08bcd	1.15±0.02bcd
LPO	10±0.98	21±0.88a	18±0.57ab	13±0.57bcd	11±0.5bcd	11.67±0.8bcd	11.3±0.87bcd	8.3±0.3bcdefg	8.6±0.8bcdefg
OH*	5.4±0.3	13±0.88a	9.7±0.3ab	7.4±0.29abc	8±0.57abc	6.2±0.39abcde	6.7±0.37abcde	5.9±0.17bcdefg	5.7±0.17bcdefg
Units: 1. H2O2- µmol/ming/mg protein
2. OH*-µmol/ming/mg protein
3. LPO-nmoles MDA formed/min/mg protein

**Table 2:** Effect of E.officinalis and Z.officinale on oxidative stress (H2O2) and lipid peroxidation (LPO) in the liver of lead-arsenic-induced toxicity in adult male rat. Each bar represents mean ± SEM of 6 animals. Significance at p< 0.05, a-compared with control, b-compared with Lead-Arsenic toxicity, c-compared with Lead-Arsenic toxicity rats treated with 60mg E.officinalis; d- compared with Lead-Arsenic toxicity rats treated with 120mg E.officinalis; e-compared with Lead-Arsenic toxicity rats treated with 60mg Z.officinale; f-compared withLead-Arsenic toxicity rats treated with 60 E.officinalis+60mg G. officinale.

Parameters	Group I	Group II	Group III	Group IV	Group V	Group VI	Group VII	Group VIII	Group IX
H2O2	2.03±0.21	4.7±0.07a	3.3±0.12ab	2.43±0.17abc	3±0.08abd	1.6±0.08abcd	1.4±0.08abcd	1.34±0.03abcd	1.36±0.05abcd
LPO	12±1.15	31±2.02a	24±2.0ab	19±0.57abc	17±1.15abc	13±0.57abcd	12±0.57bcd	11±0.8bcd	13±0.52bcde
OH*	8±0.5	20±0.5a	15±0.5ab	11±0.57abc	12±0.50abc	9.5±0.28abcde	9±0.5bcde	8±0.57bcde	8.3±0.8bcde
Units: 1. H2O2- µmol/ming/mg protein
2. OH*-µmol/ming/mg protein
3. LPO-nmoles MDA formed/min/mg protein

**Table 3:** Effect of E.officinalis and Z.officinale on antioxidant enzymes in the kidney of lead-arsenic-induced toxicity in adult male rat. Each bar represents mean ± SEM of 6 animals. Significance at p< 0.05, a-compared with control, b-compared with Lead-Arsenic toxicity, c-compared with Lead-Arsenic toxicity rats treated with 60mg E.officinalis; d- compared with Lead-Arsenic toxicity rats treated with 120mg E.officinalis; e-compared with Lead-Arsenic toxicity rats treated with 60mg Z.officinale.

Parameters	Group I	Group II	Group III	Group IV	Group V	Group VI	Group VII	Group VIII	Group IX
CAT	40±1.7	20±0.8a	26±2.3ab	32±1.4abc	33±1.7abc	41±0.8bcde	39±0.3bcf	40±1.15bcde	42±1.4bcde
GPx	32±1.4	16±1.15a	21±0.6ab	27±1.4bc	32±1.15bcd	34±2.6bcd	36±2.6bcd	38±3.05bcd	34±2.08bcd
GST	25±2.3	14±1.15	17±1.4	24±1.15	22±1.15	22±0.5	24±1.4	26±1.7	28±1.52
SOD	35±2.3	16±1.15	22±1.5	31±1.3	28±2.02	32±1.15	33±1.5	36±1.8	36±3.05

**Table 4:** Effect of E.officinalis and Z. officinale on antioxidant enzymes in liver of lead-arsenic-induced toxicity in adult male rat. Each bar represents mean ± SEM of 6 animals. Significance at p< 0.05, a-compared with control, b-compared with Lead-Arsenic toxicity, c-compared with Lead-Arsenic toxicity rats treated with 60mg E. officinalis; d- compared with Lead-Arsenic toxicity rats treated with 120mg E.officinalis; e-compared with Lead-Arsenic toxicity rats treated with 60mg Z. officinale

Parameters	Group I	Group II	Group III	Group IV	Group	Group VI	Group VII	Group VIII	Group IX
CAT	65±2.8	35±2.8a	44±3.0b	55±2.8abc	53±3.5abc	61±5.7bc	61±5.7bc	64±2.9bc	61±4.4bc
GPx	54±4.2	21±1.8a	33±1.6ab	38±1.5ab	46±3.3abcd	50±2.8bcd	50±5.78bcd	48±4.48bcd	51±1.88bcd
GST	35±2.8	17±1.3 a	23±2.0 ab	30±0.8abc	29±2.0ab	35±2.8bc	34±2.3bc	37±1.5bc	36±2.3bc
SOD	55±2.9	32±1.7 a	42±1.5ab	48±1.6ab	50±2.8abc	56±1.0bcd	51±1.8bcd	55±2.8bcd	53±4.4bcd

**Figure 3:**
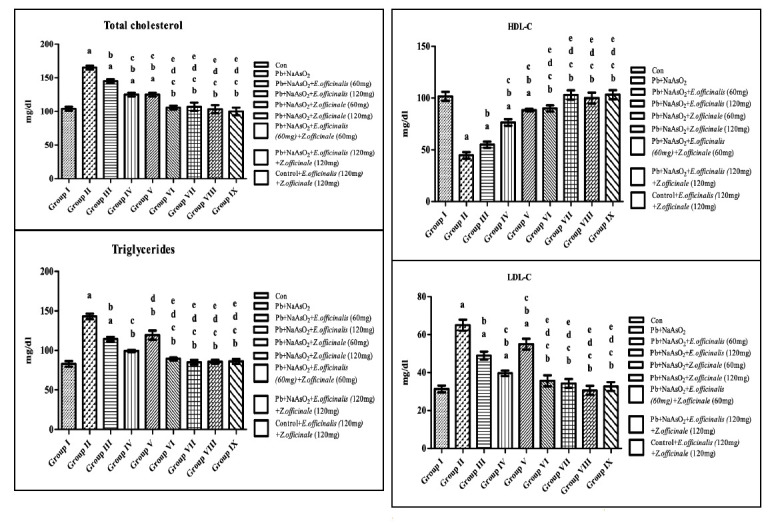
Effect of E.officinalis and Z.officinale on lipid biomarkers (TC, TG, HDL-c and LDL-c) in lead-arsenic-induced toxicity in adult male rat. Each bar represents mean ± SEM of 06 animals. Significance at p< 0.05, a-compared with control, b-compared with Lead-Arsenic toxicity, c-compared with Lead-Arsenic toxicity rats treated with 60mg E.officinalis; d- compared with Lead-Arsenic toxicity rats treated with 120mg E.officinalis; e-compared with Lead-Arsenic toxicity rats treated with 60mg Z.officinale; f-compared with Lead-Arsenic toxicity rats treated with 60 E.officinalis+60mg Z.officinale.
